# On Religious Influence in Bioethics: The Limits of Pluriversalism

**DOI:** 10.1111/bioe.13419

**Published:** 2025-04-28

**Authors:** Giovanni Spitale, Federico Germani, Brian D. Earp, Sebastian Porsdam Mann, Maide Barış, Marco Annoni, Kiarash Aramesh, Zohar Lederman, Calvin W. L. Ho, Karel Caals, Ambra D'Imperio, Marcello Ienca, Shenuka Singh, Debora Spagnolo, Nikola Biller‐Andorno

**Affiliations:** ^1^ Institute of Biomedical Ethics and History of Medicine University of Zurich Zurich Zurich Switzerland; ^2^ Centre for Biomedical Ethics National University of Singapore Singapore Singapore Singapore; ^3^ Oxford Uehiro Centre for Practical Ethics University of Oxford Oxford UK; ^4^ Center for Advanced Studies in Bioscience Innovation Law (CeBIL), Faculty of Law University of Copenhagen Copenhagen Denmark; ^5^ Faculty of Law University of Oxford Oxford UK; ^6^ Department of Medical History & Ethics Marmara University, School of Medicine Istanbul Türkiye; ^7^ Interdepartmental Center for Research Ethics and Integrity (CID Ethics) CNR Rome Italy; ^8^ The James F. Drane Bioethics Institute PennWest University Edinboro PA USA; ^9^ Department of Emergency Medicine University of Hong Kong Hong Kong Hong Kong SAR China; ^10^ Faculty of Law Monash University Melbourne Australia; ^11^ Centre for Medical Ethics and Law University of Hong Kong Hong Kong Hong Kong SAR China; ^12^ PHG Foundation University of Cambridge Cambridge UK; ^13^ Asian Bioethics Review Singapore Singapore; ^14^ Institut für Geschichte und Ethik der Medizin Technische Universität München München Germany; ^15^ Universitäre Psychiatrische Dienste Bern (UPD) Bern Switzerland; ^16^ Universitätsklinik für Forensische Psychiatrie und Psychologie (FPP) Bern Switzerland; ^17^ Laboratory of Ethics of Artificial Intelligence and Neuroscience, Institut für Geschichte und Ethik der Medizin Technische Universität München München Germany; ^18^ College of Humanities EPFL Lausanne Switzerland; ^19^ Discipline of Dentistry, School of Health Sciences University of KwaZulu‐Natal Durban South Africa; ^20^ Center for Mind and Brain Sciences University of Trento Rovereto Italy

**Keywords:** bioethics, global bioethics, pluralism, pluriversalism, religion, religious influence, secularism

## Abstract

The World Congress of Bioethics held in Qatar in 2024 (WCB 2024) sparked controversy around the role of religion in bioethics, highlighting the need for critical discussions. During the congress, there was a strong push for incorporating religious values into bioethical discourse, raising questions about the validity and implications of such an approach. This paper examines the influence of religious thought on bioethical discussions, and the ongoing debate over the role of religious perspectives in this field. Here, we explore Jecker and colleagues’ *pluriversal* framework, which was proposed at WCB 2024, espousing a bioethical discourse grounded in civility, respect for law, justice, non‐domination, and toleration. While the framework aims to embrace the world's cultural and religious diversity, here, we suggest that it struggles with significant ethical inconsistencies, poses challenges for pluralistic dialogue, and may be hard to reconcile with human rights. Through an analysis of Jecker's principles and their application, we discuss the difficulty of integrating conflicting religious views with ethical values and with widely accepted human rights frameworks. We then proceed to examine how and why religions might exert undue influence on bioethics, and we argue for a different future for bioethics.

## 1 The Long‐Standing Debate Over the Role of Religions in Bioethics

The World Congress of Bioethics (WCB) serves as a critical platform for discussing pressing ethical issues in medicine and science. The theme chosen for the 2024 Annual Meeting was “Religion, Culture, and Global Bioethics.” The conference took place in Qatar, a decision that stirred considerable controversy, with supporters arguing that the venue choice qualified as anti‐discriminatory, global, and inclusive [1], and opponents arguing that Qatar's stance on human rights issues, particularly in relation to sexual and gender minorities, is incompatible with genuine inclusivity as well as respect for all persons [2, 3].

WCB 2024 raised another critical issue: the significant push for accepting religious worldviews as *inherent* sources of moral values, such that various religious beliefs or precepts would be given substantive normative weight in bioethical discourse and analysis, whether or not they could be independently supported through a process of public reason. Several sessions at the conference prominently featured religious perspectives as foundational sources of moral guidance [4]. While some of the sessions engaged critically with the interplay between ethics and religion, others advocated for the straightforward and direct inclusion of religious values in bioethical deliberations, for example, by presenting them without argument or justification as though they possessed an intrinsic (if not necessarily universally recognizable) moral weight. This development raises questions about the role of religion in shaping ethical standards, if any.

In this paper, we examine the influence of religious thought on bioethical discussions and the ongoing debate over the role of religious perspectives in this field. This long‐standing and contentious issue resurfaced prominently at WCB 2024, but this time via the novel lens of ‘pluriversality.’ Pluriversality, discussed in detail below, is a concept recently defended by the bioethicist Nancy Jecker [5], who advocated for a world in which different cultures, traditions, and knowledge systems coexist without being subsumed under a singular, ostensibly universal framework (often characterized in this discourse as really being Western). The concept is often used in decolonial theory and philosophy to emphasize the importance of acknowledging and respecting the diversity of ways of knowing and being in the world [6, 7, 8].

“Religion,” as we use the term for the purposes of this paper, can encompass both individual beliefs and shared doctrines. Individual belief is personal and subjective, reflecting a person's unique spiritual experiences and interpretations [9]. In contrast, doctrine is the collective set of beliefs, moral principles, and ethical guidelines, formally established and taught by religious institutions [10]. These doctrines shape the structure and operations of religious institutions, which in turn can reinterpret and adapt doctrines to address contemporary issues and evolving societal values. In this paper, our focus is on the doctrinal and organizational aspects of religion, examining how these collective elements influence bioethical discussions and decisions, rather than on personal, individual beliefs.

Religious thought has significantly influenced ethical debates [11, 12]. Religious doctrines have particularly shaped attitudes toward life, death, and the moral implications of medical interventions [13, 14]. Religious leaders in many societies have played a crucial role in framing bioethical issues, guiding not only their followers but also influencing broader societal norms and policies [15, 16, 17, 18, 19, 20]. Catholicism, for example, has historically impacted discussions on the sanctity of life, leading to strong positions against practices such as abortion and principles such as reproductive autonomy [21]. Similarly, Islamic teachings have influenced perspectives on organ donation [22], and orthodox Jewish ethical thought has contributed significantly to debates on brain death [16].

The debate over the role of religion in bioethics is a persistent and thorny issue. Proponents of including religious perspectives in bioethics discourse argue that religions provide a rich source of moral guidance and reflect the values of many people worldwide [23]. They believe that ignoring religious viewpoints would alienate significant portions of the population and overlook important moral insights. Religious traditions, they argue, offer time‐tested ethical frameworks that can enrich bioethical discourse and provide a sense of moral continuity and stability [24, 25]. Some authors argue that contributions from religion can offer valuable insights into medical ethics, particularly in areas such as end‐of‐life care, genetic engineering, and reproductive rights [26, 27], and that including religious viewpoints can also enhance cultural sensitivity and respect for diverse moral traditions in global bioethical discussions [28]. The inclusion of these perspectives is also seen as a way to ensure that policies pertaining to bioethical issues are more inclusive and reflective of the pluralistic societies that they serve.

Over the last few decades, however, bioethical discourse has increasingly embraced secular arguments as the basis for reaching moral conclusions amid moral disagreement. Secularism can be defined as a position that rejects religious reasons as sufficient justification in public debates and opposes the appeal to religious assumptions in the governance of social institutions [29]. Defined in this sense, secularism consists of a procedure for public reasoning in pluralistic societies where there are competing visions of the good, and competing metaphysics, and yet, we must find ways to coexist peacefully through shared understandings. By integrating secularism in bioethics, bioethics is not confined by particular religious doctrines that may be incompatible with other specific religious traditions. While separating religious and ethical values on the personal level may be implausible, on a more institutional and therefore political level, secular bioethics can promote a broader dialogue that accommodates diverse viewpoints, *including* diverse religious viewpoints. At first sight, this might sound paradoxical, as secular approaches to ethics might be thought to exclude religious viewpoints by definition. However, secular ethical approaches may overcome this hurdle in different ways, for instance, by incorporating the principle of *public reason* [30, 31]. In Rawls’ political liberalism, public reason is the shared framework by which citizens justify political decisions without resorting to personal doctrines, meaning that substantive ethical claims must be justifiable in terms of premises that any rational person could accept, irrespective of their religious commitments. Religious approaches to ethics, in contrast, often advance substantive normative claims that emerge from particular religious teachings and convictions and are justifiable only on those grounds. Thus, there is a fundamental asymmetry, in terms of inclusiveness, between secular and religious approaches to ethics: whereas secular approaches can accommodate religious insights into ethics that can be justified in terms of public reason, religious approaches to ethics cannot accommodate substantive ethical claims that do not conform to religious doctrine. Similarly, this appeal to public reason obliges religious persons to translate their religious convictions into generally acceptable arguments [30, 31]. It is arguably on this basis that the drafters of the 1948 Universal Declaration of Human Rights (UDHRs) and subsequent human rights instruments were able to avoid drawing on religious doctrines as justification. The UDHR framework aims to create a common ethical ground that respects religious diversity while ensuring that ethical standards are broadly justifiable and thus truly inclusive. By operating within a secular framework, bioethics can address ethical dilemmas through shared reason and evidence on the grounds of a “reasonable pluralism” [32, 33, 34]. In contrast, religious perspectives can be exclusionary and may not always align with those protected by international human rights law [35, 36, 37]. Therefore, a secular approach facilitates broader participation in bioethical debates.

Secular critics maintain that ethical decisions should be based on publicly justifiable principles, without reliance on specific religious beliefs that not everyone shares, cautioning against the imposition of religious values on pluralistic societies, where individuals may hold diverse and sometimes conflicting moral views [11, 38]. Furthermore, critics of religious bioethics argue that religious perspectives can sometimes hinder creativity [39], scientific progress [40, 41, 42], and the development of policies that appropriately recognize individual autonomy and rights or can promote the rights, interests, or political power of particular faith groups while suppressing the rights of those who do not subscribe to the same beliefs and worldviews. For instance, strict religious views on issues like contraception and stem cell research can limit scientific exploration on grounds that are not justifiable to public reason [41]. They may also limit the availability of medical treatments, including for people who do not share the religious presuppositions behind such limits, sometimes—as in the case of the Texas ban on early abortions—with grave repercussions “in terms of trauma to families and medical cost as a result of increases in infant mortality” [43].

We do not deny that religions may include time‐tested and considerable bodies of knowledge or wisdom, while also being highly effective in motivating people to achieve certain ends that are widely recognized as morally good (e.g., acts of charity). However, they are not the only sources of knowledge or wisdom, and may also be highly motivating to some people to act in ways that are harmful or destructive (e.g., suicide bombing; religious crusades). Needless to say, some non‐religious worldviews, including misguided or dogmatic approaches to secularism, can be destructive as well. The challenge, therefore, is to listen to the moral insights offered by both religious and non‐religious stakeholders while ensuring that ethical frameworks developed within bioethics can be justified in terms of public reason [20].

## 2 The Call for Religious (Bio)Ethics at WCB 2024

At WCB 2024, as noted previously, numerous presentations focused on the intersection of ethics and religion. Among many, here, we engage with two prominent presentations, those by Joseph Tham [44] and Aasim Padela [45], both of whom addressed the marginalization of religious viewpoints in bioethics and advocated for a more inclusive approach that incorporates religious ethical frameworks. Here, we choose these two presentations because of Tham's and Padela's roles and influence in shaping the discourse of Catholic and Islamic bioethics, respectively.

At WCB 2024, Tham argued that the marginalization of religion in bioethics leads to a narrow and incomplete understanding of ethical issues. Tham contended that the sidelining of religious viewpoints deprives bioethical discussions of the rich moral insights and values that these perspectives offer. He emphasized that religious traditions have long histories of grappling with moral dilemmas and that their exclusion from bioethical discourse weakens the field, leading him to advocate for the inclusion of religious values to enrich bioethical dialogue. Here, we understand Tham's definition of “religious values” as values considered to be morally good from within a particular religious worldview, but that is not necessarily also recognizable as a value by people who have other religious worldviews, or no religion. Tham referenced the ‘dialogue’ between Ratzinger and Habermas, which was said to highlight the complementary roles of religion and reason in ethical discourse [46]. Tham focused on the exclusion of religion from bioethics, due to secularism, and did not discuss or even mention the constraints that should be applied to religious influence. However, even Ratzinger, in his dialogue with Habermas, argued that religion and reason should mutually inform and constrain each other.

Padela argued that Islamic bioethical perspectives are often overlooked or underrepresented in mainstream bioethical discourse. Padela called for an academic home where Islamic bioethics can be rigorously developed and integrated into the broader field. He emphasized the need for bioethical deliberations to reflect the moral and ethical teachings of Islam, which he believes can offer valuable insights into contemporary bioethical issues. Padela's vision includes the creation of dedicated academic programs, research centers, and scholarly networks focused on Islamic bioethics [47, 48]. However, Padela did not engage with a deeper question: what does the “Islamic Bioethics” framework offer that is different from Islamic jurisprudence, which also produces moral judgments based on religious rulings for practicing Muslims? Will this framework help tackle bioethical issues when dealing with Muslim patients in a non‐Muslim country or will it make it more challenging for Muslims (who consider themselves secular) living in a Muslim country? How will this framework serve the bioethics discipline? If the main goal is to inform policies and decisions about the sensitivities and priorities of Muslim patients, this endeavor already aligns with the values of secular bioethics as well. So why do we need it if such a framework is already included within the existing one? However, if it aims to make policies and decisions more restrictive in light of religious teachings specific to a certain religion, then we must question the proposed framework and such an intention from the very beginning. Furthermore, given the diversity in religious beliefs and views within geographical settings, how does an Islamic bioethics paradigm align with other religious or secular beliefs within these settings? How will the Islamic bioethics framework be applied in countries that have diverse populations with different religions? Will this then require an Islamic framework, a Christian framework, a Hindu framework, and so on within the same country/region?

Padela's call for a more prominent role of Islamic ethics in bioethics closely mirrors Tham's arguments regarding the marginalization of religious perspectives: both highlighted the importance of incorporating religious viewpoints to enrich bioethical discussions; both argued that the exclusion of religious perspectives leads to an incomplete and biased understanding of ethical issues; and both advocated for an approach that acknowledges the moral contributions of religious traditions.

Insights from religious views are not necessarily incompatible with secular and evidence‐based bioethics: moral insights from religious teachings that can be justified through public reason are already welcome into these frameworks. Moreover, secular bioethical approaches, by their own principles, must consider (and in general are already considering) the empirical reality that religious beliefs shape the values and motivations of much of the global population.

Much depends on the specific claim being made. If the claim is that, in practice (as a sociological matter), the genuine, publicly justifiable moral insights from various religious perspectives have not been sufficiently incorporated into the field, or that religious bioethicists using public reason are unfairly excluded from key bioethics debates, that would require examining specific evidence. However, if the idea is that distinctively *Islamic, Christian Catholic, or other religious* moral insights—that is, purported moral insights that *cannot* be justified in terms of principles that members of different religions, or no religion, can accept—should be allowed to stand as unquestioned premises in bioethical arguments whose conclusions are meant to apply outside the religion, then we would object.

Regarding the latter possibility, we caution that religions may aim to regain dominance over a field that they perceive as overly secularized, potentially overshadowing the principles of secular bioethics. While secular bioethics is inherently compatible with diverse religious views that can be rationally justified across different belief systems, religious views may not always align with the pluralistic and evidence‐based nature of secular bioethics.

## 3 A “Pluriversal” Framework and Its Limits

For many people, religions are a fundamental aspect of their value system and identity [49], and surely, recognizing the significance of religious views and perspectives in bioethical discourse sometimes allows for a more multifaceted understanding of moral issues. By including religious perspectives, bioethics can acknowledge and address the specific moral concerns of populations that hold religious beliefs [28]. These reasons highlight why bioethics needs to be pluralistic in the sense that it can consider various legitimate arguments of different origins in an appropriate manner. That said, at WCB 2024, Nancy Jecker proposed to go beyond pluralism via what she called a “pluriversal framework for ethics” [50]. The framework, with minor adjustments, was recently published [5]. In the following, unless otherwise specified, we refer to the WCB 2024 presentation, as at the time of writing this paper, that was the only available source.

Pluriversalism, rooted in decoloniality, challenges the dominance of Western ethical paradigms and seeks to reclaim marginalized cultural and intellectual spaces [6, 7, 8]. It aims to dismantle colonial power structures (often justified by religion) [51, 52] and promote the coexistence of multiple, equally valued epistemologies. Advocates like Grosfoguel critique what is taken to be a Western form of universalism for detaching knowledge from specific contexts and perpetuating epistemological racism by prioritizing white, male perspectives [7]. Pluriversalism claims to offer an alternative by promoting dialogues that respect diverse worldviews, aiming to “decolonize knowledge” and acknowledge contributions from all cultures [7].

According to Jecker, in a world characterized by cultural and religious diversity, a pluriversal approach to bioethics acknowledges and respects the plurality of moral perspectives better than a *universal* one. For Jecker, pluralism is not enough to ensure a culturally sensitive ethical discourse. The framework that she presented at WCB 2024 comprises five principles, which she listed as follows (direct quotes are from Jecker's slides):

- “Civility is the ethical requirement to engage others with respect.” According to Jecker, civility demands that individuals engage in bioethical discussions with mutual respect, recognizing the dignity of all participants and fostering constructive dialogue. This principle ensures that all voices in bioethical discourse are heard and valued.
- “Respect for law holds that the resolution of disputes should generally respect the law of the land.” According to Jecker, this principle emphasizes the importance of adhering to legal frameworks when resolving bioethical disputes, ensuring that decisions are made within the bounds of established laws. Respect for law helps maintain order and legitimacy in bioethical deliberations.
- “Justice involves giving people their due.” In Jecker's formulation, justice requires that individuals receive fair treatment and that their rights are upheld in bioethical deliberations, ensuring equity and fairness in ethical decisions. This principle is fundamental to addressing inequalities and promoting social justice.
- “Non‐domination prohibits others’ arbitrary and controlling influence.” This principle seeks to prevent any form of arbitrary control or domination, promoting autonomy and protecting individuals from coercive influences.
- “Toleration refers to freedom from bigotry or undue severity in judging others, forbearance.” According to Jecker, toleration encourages openness to diverse perspectives and the avoidance of severe or unjust judgements, fostering an environment where different viewpoints can be considered and respected.

These principles remained largely unchanged in the recently published version. Civility was expanded to include active listening and a willingness to learn; respect for law was renamed “change from within”—a mostly cosmetic change, as the new version posits, in line with the previous formulation, that “even when harms occur, and practices and policies require reform, it is often more effective, as well as ethically preferable, to identify and support drivers of change from within” [5]; and justice was broadened to include a subjective element (justice must also be felt as fair). Here, we critique Jecker's framework, focusing on its principles as well as on broader concerns about its ethical coherence and justification. We argue that these principles, depending on how they are interpreted, are either benign truisms that are also accepted within secular approaches to pluralism or they fail to address key ethical conflicts, particularly in legitimizing oppressive practices and insufficiently protecting human rights. Moreover, in some cases, they appear to come into conflict, not only with certain commitments of secularism but also *other* principles within Jecker's own account of pluriversalism, making the account potentially incoherent.

### 3.1 On Civility

We argue that on one interpretation, Jecker's call for civility is unobjectionable, but also unoriginal, as it is also a commitment of secular pluralism, while on another interpretation, it could be highly problematic, and indeed in tension with other principles in her own pluriversalist account. To take the first point first: civility, as defined by Jecker, is not in any way distinctive of a “pluriversal” approach to bioethics. The ethical requirement to engage others with due respect is even a plausible candidate for a universally recognized moral principle, and, in any case, it is one that is justifiable by public reason and therefore compatible with existing secular approaches to bioethics. But if Jecker is simply endorsing the moral truism that people should treat one another with respect, it is striking that she chooses the term ‘civility’ to express this. While civility ostensibly promotes polite social conduct, its colonial undertones^1^ and history of exclusion make it a contentious and problematic term. Civility, moreover, may not always be the most appropriate or ethically justified mode of behavior, even from *within* a pluriversal framework. This is because other principles in the framework, such as non‐domination, might sometimes require, or at least excuse, an “uncivil” response: for example, if there is no other way for an oppressed or silenced group to make its voice heard. Thus, according to some schools of thought that, like pluriversalism, focus on the need to resist and oppose arbitrary domination, civility is not always called for [53, 54]. Thus, Jecker's call for “civility” is either a restatement of an uncontroversial and plausibly universally accepted moral principle (albeit one that, undoubtedly, is not always adhered to in practice) or it is a proposal that may not withstand scrutiny even from within explicitly anti‐oppressive frameworks.

### 3.2 On Respect for Law

Jecker's principle of “respect for law” emphasizes adherence to the legal frameworks of a given society. The same dilemma as above applies here. While the renaming to “change from within” in the latest formulation of the pluriversal principles softens the endorsement of a blanket respect for law [5], the underlying concerns regarding the potential conflict between respecting law and addressing oppression or injustice still apply. For example, even when promoting change from within, one must still grapple with which laws are considered legitimate, how to balance respect for cultural or legal pluralism with universal human rights, and how to navigate conflicts between oppressive local laws and international standards. As for the first formulation of this principle, that is, “respect for law,” this is stating something that almost no one (apart from, perhaps, a committed anarchist) would disagree with, or, it is saying something that is potentially incompatible with other pluriversalist principles, making the overall view incoherent. On the first point: it is a moral cliché that one should, *in general* or *all else being equal*, follow the laws of the jurisdiction that one finds oneself in. But if a law is itself unjust, oppressive, or intolerant, then it runs into direct conflict with most, if not all, of the other pluriversalist demands. Following an unjust law, for instance, might require that one violate Jecker's principle of justice; following an oppressive or intolerant law might require that one violate the principles of non‐domination or tolerance, respectively. And so on. This is hardly an academic squabble. Instead, the principle raises real concerns when oppressive regimes use or introduce laws to justify systematic violations of people's rights or to subordinate or even seek to exterminate certain groups as, for example, occurred in Nazi Germany during the Second World War. In a pluriversal framework, where each culture or society defines its own legal and ethical standards, oppressive laws—such as those criminalizing same‐sex sexual behavior, to use a current example—could be upheld under the guise of respecting cultural differences. This risks legitimizing injustice, as individuals who do not subscribe to these religious or cultural beliefs may be forced to endure oppressive practices. Moreover, it is unclear to whom this principle should be applied: to visitors to the country? Or dissenters within the country? Or both? Without being checked by universal ethical standards (i.e., standards that can be justified by public reason), this principle may inadvertently protect laws that perpetuate human rights abuses, further intensifying ethical conflicts and injustices. Finally, there is the problem of conflicts *between* different laws or legal systems, for example, between a local law and international human rights law. How are such conflicts to be sorted within a pluriversal framework that calls for us to respect the law? Is international human rights law not a legitimate form of law? If so, why are only domestic‐level, but not international, laws legitimate laws that ought to be respected?

### 3.3 On Justice

The pattern continues. If justice means fairness, or giving people their due, then who could disagree? Certainly not secular pluralists. But as before, Jecker's framework struggles to address conflicts *between* principles where practices justified by religious beliefs (or beliefs generated at the intersection of culture and religion) violate ostensibly pluriversal principles such as justice. Jecker's principle of “justice” requires fair treatment and equity, and yet, some religious doctrines treat people of different sexes or genders differently in a way that might seem patently unfair or inequitable (e.g., different standards of modesty for men and women; men, but not women, allowed to serve in leadership roles within the religion, and so on). Moreover, restrictive legislations based on contentious religious views (e.g., highly restrictive abortion bans) highlight the challenges of reconciling cultural and religious beliefs with human rights such as the rights to health and to bodily integrity of pregnant women [55, 56, 57].

### 3.4 On Non‐Domination

Jecker's opposition to arbitrary control or domination, support for autonomy, and desire to protect individuals from coercive influences is admirable. But none of this is distinctive of pluriversalism: such ideals are central to secular pluralism, especially within liberal societies. Moreover, the problem of internal incoherence arises here again, as noted previously in our critique of the “Respect for law” principle. For example, regardless of a country's laws, women seek abortions at similar rates: about 37 out of 1000 when it is illegal and 34 out of 1000 when it is permitted [58, 59]. The critical difference lies in the risk to women's health, as unsafe, clandestine abortions are a significant cause of maternal deaths globally [60]. This demonstrates a direct clash with Jecker's principle of non‐domination, since strict abortion bans exert coercive control over women's bodies and decisions.^2^ Finally, insofar as any religious doctrines or practices violate the principle of non‐domination (e.g., of men over women, or parents over their children), even if these are not officially enshrined in law, they may come into conflict not only with the principle of non‐domination but also the principle of toleration, discussed next. In short, does pluriversalism require that we should tolerate practices that violate other commitments of pluriversalism, such as non‐domination?

### 3.5 On Toleration

Similar to civility, justice, and so on, the way Jecker conceives of toleration appears to be entirely unobjectionable and not in any way distinctive to a “pluriversal” framework. Secular public reason is enough to establish the moral values of “freedom from bigotry or undue severity in judging others […] openness to diverse perspectives [and] fostering an environment where different viewpoints can be considered and respected.” [5, 50] The problem, again, is internal inconsistency in Jecker's view: What shall we say about religious perspectives that are themselves intolerant or bigoted, for example, toward sexual or gender minorities? Should we tolerate intolerance? This is a classic puzzle within liberal thought, one already covered in John Locke's Letter Concerning Toleration, which maintained that toleration had boundaries: the state was not obligated to tolerate doctrines that could undermine civil society or impinge on the rights and freedoms of others. In it, he implicitly distinguishes among the political obligation to tolerate the private religious views of individuals, the private domain in which one holds one's own religious views, and the professional obligation of the magistrate, who must set aside personal beliefs when ruling on the actions of those whose religious views differ from his own. Locke's insight supports the notion that toleration is not an unconditional acceptance of all beliefs—particularly those that propagate bigotry or hostility [62]. Much more could be said about it than can possibly be covered here, but on various plausible ways of resolving it, principles of toleration such as those defined and defended by Jecker can only truly be upheld when perspectives and practices that are themselves intolerant or bigoted—whether associated with religious doctrines or institutions or otherwise—are actively resisted.

Taking these critiques together, a significant drawback of the pluriversal framework is that it can lead to ethical incoherence or other internal inconsistencies. But there is also an even deeper concern about incoherence that lies at the heart of pluriversalism itself: namely, that it is self‐undermining. If different and potentially incompatible perspectives are deemed equally valid, then perspectives that oppose pluriversalism are just as valid as those that support it, suggesting that there is no particular reason to adopt a pluriversal standpoint, after all. Moreover, from a practical perspective, it becomes challenging to make clear ethical decisions in an interconnected world where choices can have cross‐cultural impact (e.g., on matters of environmental justice, or technology governance) where universal standards, or at least the possibility to discuss them, are increasingly necessary. How can we determine which values should be included in a pluriversal framework, especially considering that even within an individual pluriversal collective, we can identify multiple distinct pluriversal sub‐worlds? If the idea is that religious frameworks, in particular, should be given greater weight, how should we define what makes a particular religious institution a serious, legitimate stakeholder? Is it about how old the religion is, or is it about how widely held are the beliefs associated with that religion?

If we advocate for pluralism, which is arguably essential, we must also establish a common ground where different religions, traditions, and cultures can engage in meaningful dialogue and a shared standpoint from where they can be scrutinized and critiqued. This common ground can be guaranteed by public reason and by human rights, for example, as attempted in the Universal Declaration of Human Rights [63]. Human rights are part of humankind's intellectual heritage and have been described as the closest thing we have to the outcome of a global ethical discourse [64]. The drafting of the Universal Declaration of Human Rights and subsequent human rights instruments involved input from different political systems, states, and institutions across the world; the International Bill of Rights has been ratified by more than 170 of the 193 UN member states. They have been influenced and endorsed by, and therefore belong to, humanity across race, gender, nationality, religion, and ethnicity [65]. In the past century, many authoritarian regimes, including both religious and ostensibly non‐religious (e.g., cult‐of‐personality) dictatorships, tried to justify the violation of human rights by calling those rights Western and alien concepts and by appealing to what they call local principles and values [66]. By grounding bioethical discourse in universally acceptable human rights and reasons, we can ensure that ethical discussions are inclusive, rational, and respectful of diverse perspectives (including religious perspectives) while maintaining clear ethical standards. This approach allows for the accommodation of pluralistic views without compromising the possibility of a meaningful dialogue. The pluriversal alternative, we contend, is not applicable. Even if it were, it would fragment humanity into an archipelago of morally isolated atolls, leaving us adrift in a sea of unreachable islands à la “cuius regio, eius religio.”^3^

## 4 Defining the Limits of Religious Influence in Bioethics

Bioethics undeniably owes a large debt to moral theology ([67], 113–24). While recognizing such historical contributions and while valuing religious perspectives as a relevant element in the worldview of a vast amount of humanity [49], oftentimes relevant in shaping decisions in matter of healthcare [28], we believe that it is important to establish clear boundaries that delineate the extent of religious influence within contemporary bioethical discourse. To do so, it is crucial to recognize that bioethical considerations span at least three distinct moral realms: political, professional, and personal. Public reason, as advocated by Rawls, should guide political decision‐making and policy formulation, ensuring that ethical deliberations are justifiable to all citizens. Professional ethics must be informed by the specialized standards and commitments of the field. Personal values and commitments shape the choices that individuals make in their private lives [68]. Understanding and considering these limits ensure a framework that accommodates diverse viewpoints while safeguarding the primacy of ethical considerations relevant to all and not just to members of a particular religious tradition.

For the sake of the argument, let us imagine that we want to include religious values in bioethics. What would this inclusion mean in practice? And whose values or priorities should be considered? There are hundreds of religious traditions. How do we adjudicate between so many traditions? Does incorporating religious values into bioethics mean consulting religious scholars for their opinions on ethical issues? Or does it involve considering the perspectives of religious clinicians who, in line with their beliefs, refuse to perform certain procedures, such as elective abortions? Alternatively, should it focus on providing necessary support to patients who, for various reasons, feel conflicted that their medical decisions, like voluntary abortion, go against their religious values? Simply stating that religious values should be included in bioethics is superficial without addressing these practical complexities [69].

The need to define the limits of religious influence in bioethics relevantly extends to public policies and legal frameworks: while religious institutions have a right to voice their perspectives, the establishment and enforcement of public policies and laws should be guided by secular principles that guarantee the space for a Rawlsian reasonable pluralism [32, 33, 34] and prioritize the well‐being of society as a whole, a position also defended by Ratzinger ([46], chap. 2). This ensures that biopolitics [70, 71, 72] are not unduly influenced by religious doctrines to the detriment of human rights.

One crucial issue of religious influence, which needs to be limited, lies in the fact that religions tend to constrain the role of extra‐doctrinal values, for example, of individual autonomy: in a pluralistic society, individuals should generally have the right to make decisions about their own bodies and health in alignment with their own values, which may or may not be grounded in religious beliefs—insofar as this is consistent with the rights of others, which can justify interventions impacting autonomy, such as vaccine mandates, quarantines, compulsory schooling for children, restrictions on second‐hand smoke, and the like. For instance, if abortion is permitted by law, it does not compel individuals who consider abortion immoral, based on their personal beliefs, to undergo or perform the procedure. This exemplifies a bioethically relevant policy that upholds reasonable pluralism. Conversely, laws that either mandate abortion (e.g., for certain conditions due to eugenic objectives) or completely prohibit it may be aligned with a pluriversal approach to bioethics, but fail to ensure a reasonably pluralistic space, as they intrude upon an area that inherently involves profoundly personal, complex, and often painful decisions.

## 5 Conclusion

In this manuscript, we have explored the intersection of religion and bioethics. We argued that religious views can and should contribute to ethics, but without any claims of inherent moral value beyond what can be justified to those outside the religious framework (especially if the conclusions of the religious analysis are expected to apply to outsiders or to internal dissenters). Religious views may offer valuable theoretical and contextual insights, but they should not be granted inherent moral force. Instead, they should be critically examined and integrated with other perspectives to inform ethical reasoning—just as we do for all other components of an ethical argument.

In this paper, we contended that bioethics as a discipline requires *some* degree of universality, at least to the extent that it is to engage with problems that cross borders, or that may even have global significance (e.g., climate change). After all, some sort of common language is required to get such discussions off the ground, ideally in a mutually respectful way [73], that enables and fosters the flourishing of collaborative human relationships that are needed, given the complex global challenges that we are facing [74]. In part, this must involve an attempt to identify or formulate ethical standards based on common values, rights, or principles that can apply across cultures and religions. Pluriversality, by contrast, allows each culture and religion to prioritize its own ethical standards, which challenges the need for a shared set of values that facilitate dialogue in bioethics. We consider some degree of universality a *conditio sine qua non* for dialogue in bioethics, rather than an obstacle to dialogue, providing a foundational framework for meaningful exchange between diverse perspectives. Therefore, here, we argue that true pluralism stands in antithesis to pluriversalism: it is crucial to distinguish between pluralistic and pluriversal approaches, as they carry different implications for the integration of diverse ethical viewpoints.

As the atheist philosopher Norberto Bobbio stated, “[t]he relevant difference for me is not between believers and non‐believers, but between thinkers and non‐thinkers; that is, between those who reflect on various questions, and the indifferent who do not reflect.” This quote, popularized by Carlo Maria Martini, a catholic cardinal and archbishop [75], underscores the importance of reflective thought in ethical discourse, transcending mere belief systems. By incorporating these elements, we advocate for a bioethics discipline that is empirically informed, theoretically robust, and inclusively pluralistic, capable of addressing the complex ethical challenges of our diverse world.

## Conflicts of Interest

The authors declare no conflicts of interest.

## Data Availability

The authors have nothing to report.

## Footnotes

1 It is intriguing that Jecker uses the concept of “civility” in her discourse. “Civility” traces back to the Latin term “civis,” meaning “citizen,” that is, “not a barbarian.” It unveils a subtle irony when one considers that the term “civility” has often been used to impose a standard of behavior that reflects the values and norms of colonial powers, thereby marginalizing and devaluing the customs and practices of colonized peoples [59].

2 The WHO also recognizes unsafe abortion (a direct consequence of abortion bans) as a barrier to achieving several SDGs (Sustainable Development Goals) [61, 74], and therefore as a global concern. Abortion bans or restrictive legislations based on religious views highlight the challenges of reconciling cultural and religious beliefs with universal ethical principles: in a pluriversal perspective, there is no space for “global concerns” by definition.

3 “Cuius regio, eius religio” (“Whose realm, his religion,” meaning that the subjects should follow the religion of their ruler) is a Latin expression that gained significant importance during the Protestant Reformation and in the centuries that followed. It indicates the obligation of subjects to conform to the confession of the prince of their state, whether Protestant or Catholic. This phrase is commonly understood in reference to European history of the 16th and 17th centuries.

## References

[1] N. S. Jecker , V. Ravitsky , M. Ghaly , J. C. Bélisle‐Pipon , and C. Atuire , “Proposed Principles for International Bioethics Conferencing: Anti‐Discriminatory, Global, and Inclusive,” American Journal of Bioethics 24, no. 4 (2024): 13–28, 10.1080/15265161.2023.2232748. 37549186

[2] C. M. Klugman , “I'm Not Welcome There: Why I Am not Attending IAB 2024,” American Journal of Bioethics 24, no. 4 (2024): 34–36, 10.1080/15265161.2024.2308135. 38529979

[3] U. Schuklenk , “The International Association of Bioethics Failed Its Rosa Parks Moment,” American Journal of Bioethics 24, no. 4 (2024): 32–34, 10.1080/15265161.2024.2308125. 38529991

[4] WCB , “Agenda for 17th World Congress of Bioethics,” 2024c, https://wcb.cilecenter.org/print-agenda/wcb?lang=en.

[5] N. S. Jecker , C. A. Atuire , V. Ravitsky , M. Ghaly , V. Vaswani , and T. C. Voo , “Religion Welcome Here: A Pluriversal Approach to Religion and Global Bioethics,” Journal of Bioethical Inquiry (2024), 10.1007/s11673-024-10410-7. PMC12263729 39680332

[6] R. Grosfoguel , “Hacia un pluriversalismo transmoderno decolonial,” Tabula Rasa no. 9, no. December (2008): 199–215.

[7] R. Grosfoguel , “Decolonizing Western Uni‐Versalisms: Decolonial Pluri‐Versalism From Aimé Césaire to the Zapatistas,” TRANSMODERNITY: Journal of Peripheral Cultural Production of the Luso‐Hispanic World 1, no. 3 (2012): 88, 10.5070/T413012884.

[8] W. D. Mignolo and C. E. Walsh , On Decoloniality: Concepts, Analytics, Praxis (Duke University Press, 2018), 10.1215/9780822371779.

[9] R. Horton , “A Definition of Religion, and Its Uses,” Journal of the Royal Anthropological Institute of Great Britain and Ireland 90, no. 2 (1960): 201–226, 10.2307/2844344.

[10] S. E. Guthrie , “Religion: What Is It?,” Journal for the Scientific Study of Religion 35, no. 4 (1996): 412–419, 10.2307/1386417.

[11] J. H. Evans , “The Social Context of Religion in the Jurisdictions of Bioethics,” American Journal of Bioethics 20, no. 12 (2020): 1–4, 10.1080/15265161.2020.1838160. 33215978

[12] T. Stuart‐Buttle , “Introduction.” in From Moral Theology to Moral Philosophy: Cicero and Visions of Humanity From Locke to Hume, eds. Tim Stuart‐Buttle (Oxford University Press, 2019), 10.1093/oso/9780198835585.003.0007.

[13] C. M. Messikomer , R. C. R. Fox , and J. P. Swazey , “The Presence and Influence of Religion in American Bioethics,” Perspectives in Biology and Medicine 44, no. 4 (2001): 485–508. 11600797 10.1353/pbm.2001.0069

[14] L. Turner , “Bioethics and Religions: Religious Traditions and Understandings of Morality, Health, and Illness,” Health Care Analysis 11, no. 3 (2003): 181–197, 10.1023/B:HCAN.0000005491.88004.27. 14708931

[15] K. Aramesh , “The Influences of Bioethics and Islamic Jurisprudence on Policy‐Making in Iran,” American Journal of Bioethics 7, no. 10 (2007): 42–44, 10.1080/15265160701588196. 17926222

[16] P. A. Kahn , “Bioethics, Religion, and Public Policy: Intersections, Interactions, and Solutions,” Journal of Religion and Health 55, no. 5 (2016): 1546–1560, 10.1007/s10943-015-0144-0. 26525211

[17] S. M. Modell , T. Citrin , M. Burmeister , S. L. R. Kardia , A. Beil , and J. Raisky , “When Genetics Meets Religion: What Scientists and Religious Leaders can Learn From Each Other,” Public Health Genomics 22, no. 5–6 (2019): 174–188, 10.1159/000504261. 31801151

[18] K. L. D. Phan , D. J. Doukas , and M. D. Fetters , “Religious Leaders’ Attitudes and Beliefs About Genetics Research and the Human Genome Project,” Journal of Clinical Ethics 6, no. 3 (1995): 237–246, 10.1086/JCE199506306. 8605386

[19] J. A. Zamer and D. L. Volker , “Religious Leaders’ Perspectives of Ethical Concerns at the End of Life,” Journal of Hospice & Palliative Nursing 15, no. 7 (2013): 396, 10.1097/NJH.0b013e31829cffa4.

[20] M. Buckler and B. Earp , “Circumcision as a Critical Test Case for the Value of Religious Bioethics: A Close Examination of the Writings of Rabbi Elliot N. Dorff.” in Bioethics and Religion: Exploring the Intersection, eds. K. R. Smith (Springer Nature, 2024).

[21] Congregation for the Doctrine of the Faith , “Instruction Dignitas Personae on Certain Bioethical Questions,” 2008, https://www.vatican.va/roman_curia/congregations/cfaith/documents/rc_con_cfaith_doc_20081208_dignitas-personae_en.html.

[22] S. A. Rasheed and A. I. Padela , “The Interplay Between Religious Leaders and Organ Donation Among Muslims,” Zygon® 48, no. 3 (2013): 635–654, 10.1111/zygo.12040.

[23] S. Richards , “Kingdoms, Priests and Handmaidens: Bioethics and Its Culture,” New Bioethics 28, no. 2 (2022): 152–167, 10.1080/20502877.2022.2071193. 35549843

[24] W. E. Stempsey , “Religion and Bioethics: Can We Talk?,” Journal of Bioethical Inquiry 8, no. 4 (2011): 339–350, 10.1007/s11673-011-9323-1.

[25] W. E. Stempsey , “Bioethics Needs Religion,” American Journal of Bioethics 12, no. 12 (2012): 17–18, 10.1080/15265161.2012.725358. 23215922

[26] A. Shabana , “Religious and Cultural Legitimacy of Bioethics: Lessons From Islamic Bioethics,” Medicine, Health Care, and Philosophy 16, no. 4 (2013): 671–677, 10.1007/s11019-013-9472-6. 23397216

[27] K. Bowman , “Bioethics and Cultural Pluralism,” Humane Health Care International 13, no. 2 (1997): 31–34. 16729408

[28] P. A. Singer and A. M. Todkill , “Bioethics for Clinicians: Continuing the Series,” CMAJ: Canadian Medical Association Journal = journal de l'Association medicale canadienne 163, no. 7 (2000): 833. PMC80506 11033711

[29] J. Berlinerblau , Secularism: The Basics (Routledge, 2021), 10.4324/9781003140627.

[30] J. Habermas , “Religion in the Public Sphere,” European Journal of Philosophy 14, no. 1 (2006): 1–25, 10.1111/j.1468-0378.2006.00241.x.

[31] J. Rawls , Political Liberalism (Columbia University Press, 1996).

[32] J. Rawls , “Justice as Fairness: Political Not Metaphysical,” Philosophy & Public Affairs 14, no. 3 (1985): 223–251.

[33] J. Rawls , “A Theory of Justice: Original Edition,” 2005a, http://gen.lib.rus.ec/book/index.php?md5=d2eec200ebed5373c6e2c58c7d645b5f.

[34] J. Rawls , Political Liberalism, Expanded ed. Columbia Classics in Philosophy (Columbia University Press, 2005b).

[35] L. Henkin , “Religion, Religions, and Human Rights,” Journal of Religious Ethics 26, no. 2 (1998): 229–239. 11657254

[36] P. Juviler and C. Gustafson , Religion and Human Rights: Competing Claims? (Routledge, 2016).

[37] C. McCrudden , “Religion, Human Rights, Equality and the Public Sphere,” Ecclesiastical Law Journal 13, no. 1 (2011): 26–38, 10.1017/S0956618X10000773.

[38] T. F. Murphy , “Theorizing Religion in Its Meanings for Bioethics,” American Journal of Bioethics 20, no. 12 (2020): 47–49, 10.1080/15265161.2020.1833102. 33196382

[39] Z. Liu , Q. Guo , P. Sun , Z. Wang , and R. Wu , “Does Religion Hinder Creativity? A National Level Study on the Roles of Religiosity and Different Denominations,” Frontiers in Psychology 9, no. October (2018): 1, 10.3389/fpsyg.2018.01912. 30349502 PMC6186845

[40] A. E. McGrath , Science & Religion: A New Introduction (John Wiley & Sons, 2020).

[41] C. Mooney , The Republican War on Science (Hachette UK, 2007).

[42] C. A. Russell , “The Conflict of Science and Religion,” in The History of Science and Religion in the Western Tradition, eds. G. B. Ferngren , E. J. Larson , and D. W. Amundsen (Routledge, 2000).

[43] A. Gemmill , C. E. Margerison , E. A. Stuart , and S. O. Bell , “Infant Deaths After Texas’ 2021 Ban on Abortion in Early Pregnancy,” JAMA Pediatrics 178 (2024): 784–791, June, 10.1001/jamapediatrics.2024.0885. 38913344 PMC11197445

[44] WCB , “17th World Congress of Bioethics, Sessions by Speaker (Joseph Tham),” 2024b, https://wcb.cilecenter.org/wcb#/agenda?day=2&lang=en&speakerId=86443000000956409.

[45] WCB , “17th World Congress of Bioethics, Sessions by Speaker (Aasim Padela),” 2024, https://wcb.cilecenter.org/wcb#/agenda?day=2&lang=en&speakerId=86443000000956110.

[46] J. Ratzinger and J. Habermas , Dialectics of Secularization: On Reason and Religion (Ignatius Press, 2006).

[47] A. I. Padela , “Islamic Medical Ethics: A Primer,” Bioethics 21, no. 3 (2007): 169–178, 10.1111/j.1467-8519.2007.00540.x. 17845488

[48] A. Padela , “Methodological and Discursive Considerations for Islamic Bioethics Research and Writing,” TAFHIM: IKIM Journal of Islam and the Contemporary World 16, no. 1 (2023): 1–34, 10.56389/tafhim.vol16no1.2.

[49] Our World in Data , “How Important Religion Is to People in Life,” Our World in Data, 2022, https://ourworldindata.org/grapher/how-important-religion-is-in-your-life.

[50] N. S. Jecker , “FAB/WCB Crossover Session.June 3,” 2024.

[51] N. E. Jaffary , Gender, Race and Religion in the Colonization of the Americas. Women and Gender in the Early Modern World (Ashgate, 2007).

[52] T.‐siong B. Liew and F. F. Segovia , Colonialism and the Bible: Contemporary Reflections From the Global South (Lexington Books, 2018).

[53] M. Cherry , The Case for Rage: Why Anger Is Essential to Anti‐Racist Struggle (Oxford University Press, 2021).

[54] R. Essex and L. Mainey , “Give Incivility a Chance,” Journal of Medical Ethics 49, no. 10 (2023): 679–680, 10.1136/jme-2023-109336. 37419669

[55] A. Adamczyk , “Religion as a Micro and Macro Property: Investigating the Multilevel Relationship between Religion and Abortion Attitudes Across the Globe,” European Sociological Review 38, no. 5 (2022): 816–831, 10.1093/esr/jcac017.

[56] K. M. Hedayat , P. Shooshtarizadeh , and M. Raza , “Therapeutic Abortion in Islam: Contemporary Views of Muslim Shiite Scholars and Effect of Recent Iranian Legislation,” Journal of Medical Ethics 32, no. 11 (2006): 652–657, 10.1136/jme.2005.015289. 17074823 PMC2563289

[57] M. Minkenberg , “Religion and Public Policy: Institutional, Cultural, and Political Impact on the Shaping of Abortion Policies in Western Democracies,” Comparative Political Studies 35, no. 2 (2002): 221–247, 10.1177/0010414002035002004.

[58] J. Bearak , A. Popinchalk , B. Ganatra , et al., “Unintended Pregnancy and Abortion by Income, Region, and the Legal Status of Abortion: Estimates From a Comprehensive Model for 1990–2019,” Lancet Global Health 8, no. 9 (2020): e1152–e1161, 10.1016/S2214-109X(20)30315-6. 32710833

[59] S. Singh , L. Remez , G. Sedgh , L. Kwok , and T. Onda , “Abortion Worldwide 2017: Uneven Progress and Unequal Access,” Guttmacher Institute, 2017, https://www.guttmacher.org/sites/default/files/report_pdf/abortion-worldwide-2017.pdf.

[60] L. Say , D. Chou , A. Gemmill , et al., “Global Causes of Maternal Death: A WHO Systematic Analysis,” Lancet Global Health 2, no. 6 (2014): e323–e333, 10.1016/S2214-109X(14)70227-X. 25103301

[61] WHO , “Maternal Mortality Fact Sheet.” Who.Int. April 26, 2024, https://www.who.int/news-room/fact-sheets/detail/maternal-mortality.

[62] J. Locke , “A Letter Concerning Toleration.1689,” 1689.

[63] UN General Assembly , “Universal Declaration of Human Rights,” United Nations, 1948, https://www.un.org/en/about-us/universal-declaration-of-human-rights.

[64] S. P. Mann , H. Porsdam , and Y. Donders , “‘Sleeping Beauty’: The Right to Science as a Global Ethical Discourse,” Human Rights Quarterly 42, no. 2 (2020): 332–356.

[65] J. Morsink . 1999. The Universal Declaration of Human Rights: Origins, Drafting, and Intent. University of Pennsylvania Press, https://www.jstor.org/stable/j.ctt3fhrpm.

[66] S. Cohen , “Government Responses to Human Rights Reports: Claims, Denials, and Counterclaims,” Human Rights Quarterly 18, no. 3 (1996): 517–543.

[67] J. K. Walter and E. P. Klein , The Story of Bioethics: From Seminal Works to Contemporary Explorations (Georgetown University Press, 2003).

[68] R. Rhodes , L. Francis and A. Silvers , ed., The Blackwell Guide to Medical Ethics. Blackwell Philosophy Guides 21 (Blackwell Pub, 2007).

[69] B. D. Earp , “Does Religion Deserve a Place in Secular Medicine?,” Journal of Medical Ethics 41, no. 11 (2015): 865–866, 10.1136/medethics-2015-102917. 26106091

[70] M. Foucault , “Society Must Be Defended,” 2020, https://www.penguin.co.uk/books/24073/society-must-be-defended-by-michel-foucault-ed-mauro-bertani-and-alessandro-fontana-trans-david-macey/9780241435168.

[71] M. Foucault and J. R. Carrette , Religion and Culture. Repr (Routledge, 2009).

[72] J. Bishop and F. Jotterand , “Bioethics as Biopolitics,” Journal of Medicine and Philosophy 31, no. 3 (2006): 205–212, 10.1080/03605310600712760. 16760100

[73] K. A. Appiah , Cosmopolitanism: Ethics in a World of Strangers, Reprint Edition (W. W. Norton & Company, 2007).

[74] N. Biller‐andorno , “Iab Presidential Address: Bioethics in a Globalized World – Creating Space for Flourishing Human Relationships,” Bioethics 25, no. 8 (2011): 430–436, 10.1111/j.1467-8519.2011.01927.x. 21929701

[75] C. M. Martini , “Bobbio: un ateo nel giusto,” la Repubblica, October 27, 1992, https://www.repubblica.it/spettacoli-e-cultura/1992/10/27/news/bobbio_un_ateo_nel_giusto-41739464/.

